# 7,11,15,28-Tetra­bromo-1,21,23,25-tetra­phenethyl­resorcin[4]arene cavitand–acetone–chloro­form (1/1.31/0.69) at 173 K

**DOI:** 10.1107/S1600536809006540

**Published:** 2009-02-28

**Authors:** Michael G. McKay, Holger B. Friedrich, R. Alan Howie, Glenn E. M. Maguire

**Affiliations:** a School of Chemistry, University of KwaZulu-Natal, Durban 4000, South Africa; b Department of Chemistry, University of Aberdeen, Aberdeen AB24 3UE, Scotland

## Abstract

The crystal structure of the title compound, C_64_H_52_Br_4_O_8_·1.31C_3_H_6_O·0.69CHCl_3_, is described. The structure has been reported previously [Bryant, Blanda, Vincenti & Cram (1991). *J. Am. Chem. Soc.* **113**, 2167–2172]; however, the lower data acquisition temperature results in an improved refinement model. In addition, the presence of residual acetone and (disordered) chloro­form within the mol­ecular structure of the title compound represents a new clathrate of the title compound. One half of the resorcin[4]arene cavitand mol­ecule appears in the asymmetric unit; the complete resorcin[4]arene cavitand structure was generated across a mirror plane.

## Related literature

For the synthesis of the title compound and details of the previously reported structure, see: Bryant *et al.* (1991 ▶) and Sherman *et al.* (1991 ▶). For analogous mol­ecules and synthetic precursors which illustrate the host capabilities of resorcin[4]arene cavitand mol­ecules, see: Friedrich *et al.* (2007 ▶); McKay *et al.* (2007 ▶, 2008 ▶). For the implemetation of the SQUEEZE function in *PLATON* (Spek, 2009 ▶), see Tam *et al.* (2005 ▶).
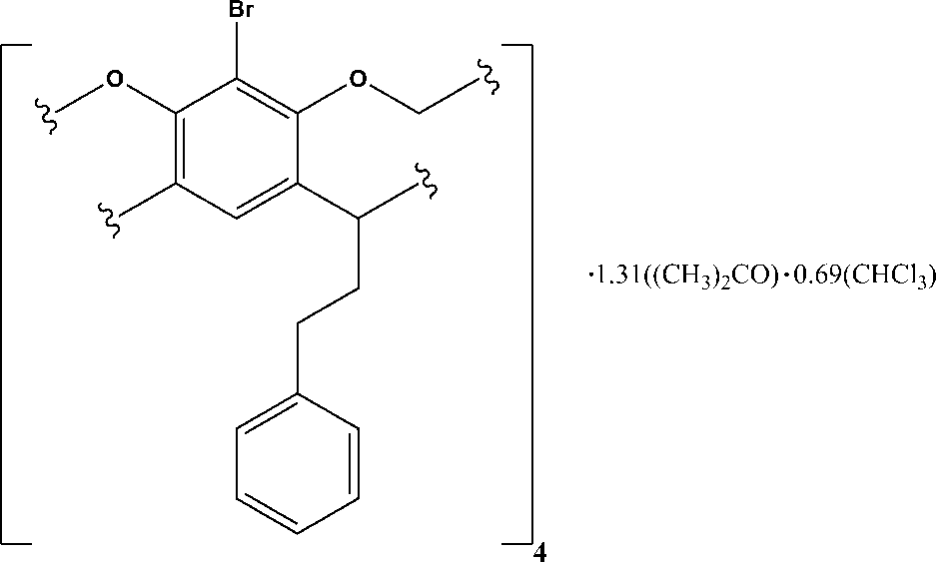


## Experimental

### 

#### Crystal data

C_64_H_52_Br_4_O_8_·0.31C_3_H_6_O·0.69CHCl_3_
*M* *_r_* = 1427.14
Orthorhombic, 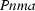

*a* = 24.7118 (18) Å
*b* = 20.4364 (13) Å
*c* = 11.9345 (8) Å
*V* = 6027.2 (7) Å^3^
*Z* = 4
Mo *K*α radiation
μ = 2.82 mm^−1^
*T* = 173 K
0.41 × 0.25 × 0.17 mm

#### Data collection

Bruker APEXII CCD area-detector diffractometer
Absorption correction: integration (*XPREP* in *SAINT-NT*; Bruker 2005 ▶)*T*_min_ = 0.391, *T*_max_ = 0.645
21376 measured reflections
5927 independent reflections
3945 reflections with *I* > 2σ(*I*)
*R*_int_ = 0.080

#### Refinement

*R*[*F* ^2^ > 2σ(*F* ^2^)] = 0.051
*wR*(*F* ^2^) = 0.138
*S* = 0.96
5927 reflections
406 parameters
H-atom parameters constrained
Δρ_max_ = 1.23 e Å^−3^
Δρ_min_ = −0.60 e Å^−3^

### 

Data collection: *APEX2* (Bruker, 2005 ▶); cell refinement: *SAINT* (Bruker, 2005 ▶); data reduction: *SAINT*; program(s) used to solve structure: *SHELXTL* (Sheldrick, 2008 ▶); program(s) used to refine structure: *SHELXL97* (Sheldrick, 2008 ▶); molecular graphics: *ORTEP-3 for Windows* (Farrugia, 1997 ▶); software used to prepare material for publication: *SHELXTL*.

## Supplementary Material

Crystal structure: contains datablocks I, global. DOI: 10.1107/S1600536809006540/hg2477sup1.cif

Structure factors: contains datablocks I. DOI: 10.1107/S1600536809006540/hg2477Isup2.hkl

Additional supplementary materials:  crystallographic information; 3D view; checkCIF report

## Figures and Tables

**Fig. 1.**
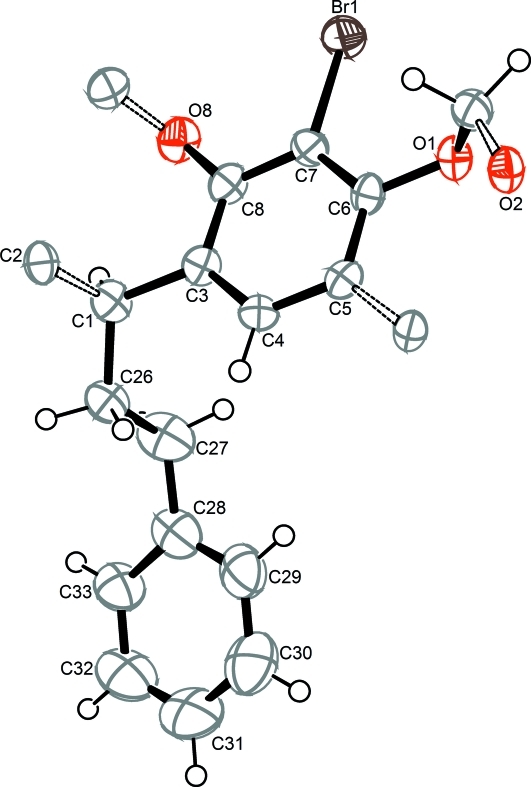
A view of one component of the cyclic tetramer. Displacement ellipsoids are drawn at the 30% probability level and H atoms are shown as spheres of arbitrary radii. Dashed bonds indicate links to the neighbouring monomer units. Selected atoms are labelled.

**Fig. 2.**
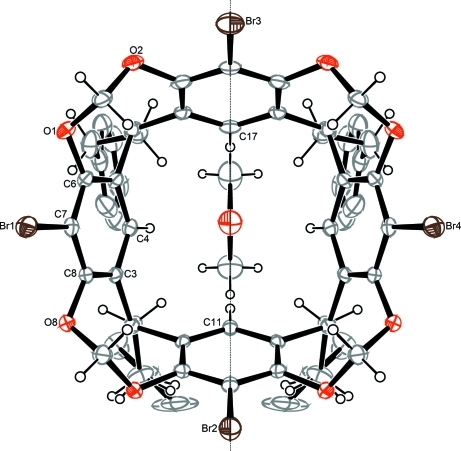
The molecular structure of the title compound, as viewed from above the molecular cavity. Displacement ellipsoids are drawn at the 30% probability level and H atoms, where shown, are drawn as spheres of arbitrary radii. The residual acetone molecule is evident. The dashed line indicates the mirror plane, which passes through C11, Br2, and C17, Br3 (as discussed).

**Fig. 3.**
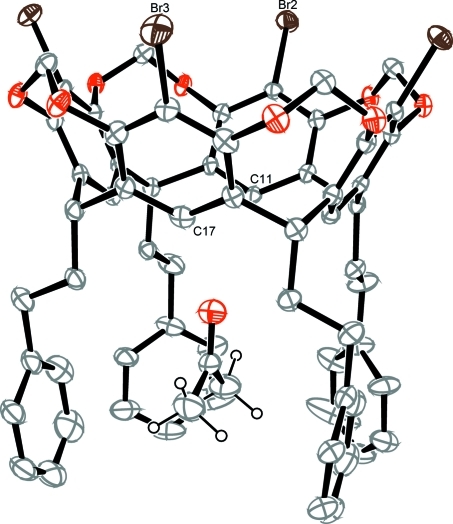
The molecular structure of the title compound viewed from side-on. Displacement ellipsoids are drawn at the 30% probability level and H atoms, where shown, are drawn as spheres of arbitrary radii. The presence of the residual acetone solvent of crystallization is evident below the molecular cavity, between the 2-phenylethyl moieties.

## supplementary crystallographic information

### Comment

The title compound (Scheme 1) is a cyclic tetramer of [4]arene moieties. The labelling scheme for one of the monomers is presented in Fig.1, and extends over the whole molecule. Dimensions are available in the archived CIF.

The title compound has been synthesized previously and its structure reported. For related literature, see Bryant *et al.* (1991) and Sherman *et al.* (1991). As such, the newly acquired data shares the same space group, similar unit-cell parameters, and similar bond lengths and angles as previously reported in the structure of Sherman *et al. *However, the lower data acquisition temperature in this case (173 K) results in an improved agreement value of 5.1%, in comparison to the previous 7.4%.

We have recently reported a number of resorcin[4]arene structures of synthetic precursors and analogues to the title compound. These exhibited the presence of residual solvents either within the confines of the molecular cavity, or on the periphery of the molecule. See Friedrich *et al.* (2007), McKay *et al.* (2007) and McKay *et al.* (2008) for related literature. The title compound exhibits the presence of residual acetone and chloroform (from crystallization), found occupying positions in the 2-phenylethyl 'feet' and cavity, respectively. This is evident in Fig. 2 and Fig. 3, the molecular structure of the title compound. In contrast, the structure of Sherman *et al.* exhibited the presence of water partially occupying positions within the feet and molecular cavity. The title compound hence represents a new clathrate of this resorcin[4]arene cavitand molecule.

However, additional solvent-related areas of electron density were located in the Fourier maps (located near inversion centres) during refinement. The related pattern suggested that the chloroform molecule, in particular, involved partial occupancy and was of a highly disordered nature. Thus, in the final refinement model, the electron density related to this disordered chloroform molecule was removed by using the SQUEEZE function of *PLATON* (Spek, 2009). This resulted in an improved refinement model. The partial contribution of the chloroform molecule was included in the molecular formula, and is further detailed in the SQUEEZE results which are appended to the CIF text. This further details the inclusion of a fractional acetone molecule (i.e 0.31, in addition to the molecule present within the 'feet') in the molecular formula. For related literature, see Tam *et al.* (2005). The use of SQUEEZE further accounts for the discrepancies seen in the calculated and reported parameters of molecular weight, density and absorption coefficient.

The asymmetric unit consist of a half of the title compound, including several atoms lying on a crystallographic mirror plane. The complete molecular structure was generated by the operation of the mirror plane upon the asymmetric unit (corresponding to one half of the tetramer) with non-hydrogen atoms C11, C12, Br2 and C17, C18, Br3 lying on the mirror plane. This plane is indicated by a dashed line in Fig. 2. The molecule was additionally completed accompanied by the molecule of residual acetone solvent discussed as present in the molecular 'feet'. Also, one of the 2-phenylethyl residues shows disorder.

### Experimental

To a solution of CH_2_BrCl (16.3 ml, 0.243 mol) and oven-dried (110 ^°^C) K_2_CO_3_ (99.25 g, 0.718 mol) in dry, degassed DMF (700 ml), bromo-octol (25.0 g, 0.0205 mol) in DMF (100 ml) was added over 1.5 h. After stirring for 24 h at room temperature under a nitrogen atmosphere, further CH_2_BrCl (16.3 ml, 0.243 mol) was added and the solution heated to 45 ^°^C. After a further 24 h, another aliquot of CH_2_BrCl (16.3 ml, 0.243 mol) was added, and the solution heated to 63 ^°^C. After 48 h at 65 ^°^C, the light brown solution was cooled to room temperature, and the K_2_CO_3_ neutralized by the addition of a 6% HCl solution. The crude product simultaneously precipitated from solution, and was collected on filtration of the neutralized reaction mixture. The cream-coloured solid was suspended in methanol and stirred for 24 h, before being filtered from the methanol and dried. The material was chromatographed on silica gel using a chloroform mobile phase, before being stirred once again in methanol, filtered and dried to give the title compound as an off-white solid. (25.90 g, 70%), mp 558–563 K dec. ^1^H NMR [CDCl_3_, 400 MHz]: d = 2.47–2.49 (m, 8 H, C*H*_2_CH_2_Ar), 2.62–2.64 (m, 8 H, CH_2_C*H*_2_Ar), 4.41 (d, 4 H, inner of OC*H*_2_O), 4.94 (t, 4 H, C*H*CH_2_CH_2_Ar), 5.95 (d, 4 H, outer of OC*H*_2_O), 7.09–7.24 (m, 24 H, Ar *H* and CH_2_CH_2_C_6_*H*_5_). ^13^C NMR [CDCl_3_,100 MHz]: d = 152.84, 141.74, 139.69, 129.25, 128.92, 126.81, 119.52, 114.41, 99.03, 38.33, 34.79, 32.89. IR (KBr): 2939*w*, 1729*w*, 1602*w*, 1469*sh*, 1452, 1415, 1299, 1234, 1091, 1057*w*, 1017, 984*sh*, 957, 790*w*, 745, 699, 586.

Crystals suitable for X-ray crystallography were grown by slow evaporation of a solution of the title compound in 1:1 chloroform:acetone.

### Refinement

Non-hydrogen atoms were first refined isotropically followed by anisotropic refinement by full matrix least-squares calculations based on F2 using *SHELXTL*. Hydrogen atoms, first located in the difference map, were positioned geometrically and allowed to ride on their respective parent atoms, with C—H bond lengths of 1.00 (CH), 0.99 (CH_2_), or 0.98 (CH_3_). They were then refined with a riding model with *U*_iso_(H) = 1.5*U*_eq_(CH_3_) and *U*_iso_(H) = 1.2*U*_eq_(*X*) for *X* = CH or CH_2_.

One of the 2-phenylethyl residues is disordered. As such, the residue was refined over two positions, with a fixed occupancy of 0.50 for atoms C21–26 and C21A-26 A. The largest residual electron density peak of 1.23 e/Å^3^ is 0.93 Å from Br3.

### Crystal data

**Table:**

C_64_H_52_Br_4_O_8_·0.31(C_3_H_6_O)·0.69(CHCl_3_)	*F*(000) = 2888
*M**_r_* = 1427.14	*D*_x_ = 1.573 Mg m^−^^3^
Orthorhombic, *P**n**m**a*	Melting point: 558 K
Hall symbol: -P 2ac 2n	Mo *K*α radiation, λ = 0.71073 Å
*a* = 24.7118 (18) Å	µ = 2.82 mm^−^^1^
*b* = 20.4364 (13) Å	*T* = 173 K
*c* = 11.9345 (8) Å	Block, yellow
*V* = 6027.2 (7) Å^3^	0.41 × 0.25 × 0.17 mm
*Z* = 4	

### Data collection

**Table:**

Bruker APEXII CCD area-detector diffractometer	5927 independent reflections
Radiation source: fine-focus sealed tube	3945 reflections with *I* > 2σ(*I*)
graphite	*R*_int_ = 0.080
φ and ω scans	θ_max_ = 26.0°, θ_min_ = 1.7°
Absorption correction: integration (*XPREP* in *SAINT-NT*; Bruker 2005)	*h* = −28→30
*T*_min_ = 0.391, *T*_max_ = 0.645	*k* = −23→20
21376 measured reflections	*l* = −14→14

### Refinement

**Table:**

Refinement on *F*^2^	Primary atom site location: structure-invariant direct methods
Least-squares matrix: full	Secondary atom site location: difference Fourier map
*R*[*F*^2^ > 2σ(*F*^2^)] = 0.051	Hydrogen site location: inferred from neighbouring sites
*wR*(*F*^2^) = 0.138	H-atom parameters constrained
*S* = 0.96	*w* = 1/[σ^2^(*F*_o_^2^) + (0.0825*P*)^2^] where *P* = (*F*_o_^2^ + 2*F*_c_^2^)/3
5927 reflections	(Δ/σ)_max_ = 0.001
406 parameters	Δρ_max_ = 1.23 e Å^−^^3^
0 restraints	Δρ_min_ = −0.60 e Å^−^^3^

### Special details

**Table:**

Geometry. All e.s.d.'s (except the e.s.d. in the dihedral angle between two l.s. planes) are estimated using the full covariance matrix. The cell e.s.d.'s are taken into account individually in the estimation of e.s.d.'s in distances, angles and torsion angles; correlations between e.s.d.'s in cell parameters are only used when they are defined by crystal symmetry. An approximate (isotropic) treatment of cell e.s.d.'s is used for estimating e.s.d.'s involving l.s. planes.
Refinement. Refinement of *F*^2^ against ALL reflections. The weighted *R*-factor *wR* and goodness of fit *S* are based on *F*^2^, conventional *R*-factors *R* are based on *F*, with *F* set to zero for negative *F*^2^. The threshold expression of *F*^2^ > σ(*F*^2^) is used only for calculating *R*-factors(gt) *etc*. and is not relevant to the choice of reflections for refinement. *R*-factors based on *F*^2^ are statistically about twice as large as those based on *F*, and *R*- factors based on ALL data will be even larger.

### Fractional atomic coordinates and isotropic or equivalent isotropic displacement parameters (Å^2^)

**Table:**

	*x*	*y*	*z*	*U*_iso_*/*U*_eq_	Occ. (<1)
C1	0.20422 (14)	0.05762 (19)	0.3969 (3)	0.0311 (9)	
C2	0.16575 (15)	0.0724 (2)	0.3167 (3)	0.0319 (9)	
C3	0.11888 (15)	0.1056 (2)	0.3475 (3)	0.0311 (9)	
C4	0.11216 (14)	0.12196 (19)	0.4597 (3)	0.0308 (9)	
H4	0.0807	0.1454	0.4813	0.037*	
C5	0.14965 (14)	0.10541 (19)	0.5412 (3)	0.0279 (8)	
C6	0.19612 (15)	0.07329 (19)	0.5079 (3)	0.0282 (8)	
C7	0.27265 (16)	0.0977 (2)	0.6250 (3)	0.0388 (10)	
H7A	0.2812	0.1286	0.5635	0.047*	
H7B	0.3064	0.0743	0.6447	0.047*	
C8	0.22623 (15)	0.1911 (2)	0.6997 (3)	0.0298 (9)	
C9	0.17120 (14)	0.19043 (19)	0.6789 (3)	0.0263 (8)	
C10	0.14312 (15)	0.1245 (2)	0.6645 (3)	0.0298 (9)	
H10	0.1640	0.0919	0.7095	0.036*	
C11	0.1443 (2)	0.2500	0.6679 (4)	0.0272 (12)	
H11	0.1066	0.2500	0.6523	0.033*	
C12	0.2540 (2)	0.2500	0.7082 (4)	0.0297 (12)	
C13	0.19362 (17)	0.0980 (2)	0.1308 (3)	0.0382 (10)	
H13A	0.2168	0.1294	0.1718	0.046*	
H13B	0.2165	0.0753	0.0751	0.046*	
C14	0.13603 (16)	0.1905 (2)	0.1238 (3)	0.0381 (10)	
C15	0.09748 (15)	0.1902 (2)	0.2090 (3)	0.0325 (9)	
C16	0.07762 (15)	0.1257 (2)	0.2572 (3)	0.0358 (10)	
H16	0.0790	0.0922	0.1961	0.043*	
C17	0.0795 (2)	0.2500	0.2504 (4)	0.0364 (14)	
H17	0.0538	0.2500	0.3096	0.044*	
C18	0.1557 (2)	0.2500	0.0836 (4)	0.0370 (14)	
C19	0.08446 (15)	0.1224 (2)	0.7067 (3)	0.0349 (9)	
H19A	0.0636	0.1582	0.6712	0.042*	
H19B	0.0677	0.0804	0.6845	0.042*	
C20	0.08183 (18)	0.1297 (3)	0.8338 (3)	0.0566 (14)	
H20A	0.1005	0.1706	0.8560	0.068*	0.50
H20B	0.1012	0.0926	0.8691	0.068*	0.50
H20C	0.1118	0.1040	0.8669	0.068*	0.50
H20D	0.0883	0.1762	0.8527	0.068*	0.50
C21	0.0221 (2)	0.1314 (5)	0.8782 (6)	0.054 (4)	0.50
C22	−0.0170 (3)	0.0885 (4)	0.8379 (6)	0.063 (3)	0.50
H22	−0.0088	0.0604	0.7767	0.076*	0.50
C23	−0.0680 (2)	0.0867 (4)	0.8870 (7)	0.074 (4)	0.50
H23	−0.0947	0.0574	0.8595	0.089*	0.50
C24	−0.0798 (2)	0.1278 (5)	0.9765 (7)	0.077 (5)	0.50
H24	−0.1147	0.1266	1.0101	0.093*	0.50
C25	−0.0408 (3)	0.1707 (5)	1.0168 (5)	0.146 (9)	0.50
H25	−0.0489	0.1988	1.0780	0.175*	0.50
C26	0.0102 (3)	0.1725 (4)	0.9677 (6)	0.066 (4)	0.50
H26	0.0369	0.2018	0.9952	0.079*	0.50
C21A	0.0304 (3)	0.1088 (5)	0.8884 (7)	0.056 (4)	0.50
C22A	0.0197 (4)	0.0446 (4)	0.9198 (7)	0.071 (3)	0.50
H22A	0.0472	0.0123	0.9138	0.085*	0.50
C23A	−0.0311 (4)	0.0276 (4)	0.9602 (8)	0.114 (6)	0.50
H23A	−0.0384	−0.0162	0.9817	0.137*	0.50
C24A	−0.0714 (3)	0.0749 (6)	0.9691 (10)	0.114 (7)	0.50
H24A	−0.1061	0.0633	0.9967	0.137*	0.50
C25A	−0.0607 (4)	0.1391 (6)	0.9376 (11)	0.28 (2)	0.50
H25A	−0.0883	0.1714	0.9436	0.335*	0.50
C26A	−0.0099 (5)	0.1561 (4)	0.8972 (10)	0.132 (8)	0.50
H26A	−0.0026	0.1999	0.8757	0.159*	0.50
C27	0.01933 (16)	0.1291 (2)	0.2997 (3)	0.0400 (10)	
H27A	0.0177	0.1593	0.3645	0.048*	
H27B	−0.0040	0.1473	0.2399	0.048*	
C28	−0.00227 (19)	0.0639 (2)	0.3342 (5)	0.0637 (15)	
H28A	0.0253	0.0409	0.3799	0.076*	
H28B	−0.0093	0.0372	0.2666	0.076*	
C29	−0.05422 (19)	0.0702 (2)	0.4016 (5)	0.0557 (13)	
C30	−0.10474 (19)	0.0702 (3)	0.3462 (5)	0.0608 (14)	
H30	−0.1059	0.0635	0.2675	0.073*	
C31	−0.1520 (2)	0.0797 (3)	0.4036 (6)	0.0733 (17)	
H31	−0.1855	0.0811	0.3647	0.088*	
C32	−0.1507 (2)	0.0872 (3)	0.5188 (7)	0.0804 (19)	
H32	−0.1835	0.0928	0.5590	0.096*	
C33	−0.1023 (3)	0.0865 (3)	0.5757 (6)	0.0841 (19)	
H33	−0.1016	0.0917	0.6548	0.101*	
C34	−0.0531 (2)	0.0778 (3)	0.5146 (5)	0.0693 (15)	
H34	−0.0195	0.0774	0.5532	0.083*	
C35	−0.0304 (3)	0.2500	0.5415 (6)	0.0545 (18)	
C36	−0.0759 (3)	0.2500	0.4528 (9)	0.095 (3)	
H36A	−0.0594	0.2500	0.3781	0.142*	
H36B	−0.0984	0.2892	0.4616	0.142*	
C37	−0.0438 (3)	0.2500	0.6615 (6)	0.075 (2)	
H37A	−0.0105	0.2500	0.7063	0.112*	
H37B	−0.0650	0.2108	0.6790	0.112*	
O1	0.23403 (10)	0.05210 (13)	0.5868 (2)	0.0325 (6)	
O2	0.25454 (10)	0.13379 (13)	0.7197 (2)	0.0333 (6)	
O3	0.17310 (11)	0.05159 (14)	0.2073 (2)	0.0391 (7)	
O4	0.15316 (11)	0.13257 (16)	0.0747 (2)	0.0425 (7)	
O5	0.01602 (19)	0.2500	0.5104 (4)	0.0638 (13)	
Br1	0.268387 (17)	0.01324 (2)	0.35408 (3)	0.04481 (16)	
Br2	0.32890 (2)	0.2500	0.73692 (6)	0.0501 (2)	
Br3	0.20877 (3)	0.2500	−0.02924 (5)	0.0641 (2)	

### Atomic displacement parameters (Å^2^)

**Table:**

	*U*^11^	*U*^22^	*U*^33^	*U*^12^	*U*^13^	*U*^23^
C1	0.033 (2)	0.023 (2)	0.037 (2)	0.0032 (17)	0.0034 (16)	−0.0012 (18)
C2	0.041 (2)	0.027 (2)	0.0282 (18)	−0.0036 (18)	−0.0019 (16)	−0.0046 (18)
C3	0.038 (2)	0.027 (2)	0.0288 (18)	−0.0046 (17)	−0.0025 (15)	−0.0052 (17)
C4	0.0304 (19)	0.027 (2)	0.035 (2)	−0.0039 (17)	0.0025 (15)	−0.0022 (18)
C5	0.0351 (19)	0.021 (2)	0.0276 (18)	−0.0087 (17)	0.0011 (15)	−0.0002 (16)
C6	0.0358 (19)	0.022 (2)	0.0271 (17)	−0.0018 (17)	−0.0028 (15)	0.0013 (16)
C7	0.039 (2)	0.041 (3)	0.037 (2)	0.007 (2)	0.0006 (17)	−0.002 (2)
C8	0.038 (2)	0.030 (2)	0.0212 (16)	0.0028 (18)	−0.0021 (14)	0.0006 (17)
C9	0.0364 (19)	0.026 (2)	0.0166 (15)	−0.0031 (17)	0.0021 (14)	−0.0026 (16)
C10	0.037 (2)	0.028 (2)	0.0252 (18)	−0.0012 (17)	0.0009 (15)	0.0019 (17)
C11	0.030 (3)	0.027 (3)	0.024 (2)	0.000	0.004 (2)	0.000
C12	0.028 (3)	0.031 (4)	0.030 (3)	0.000	−0.002 (2)	0.000
C13	0.045 (2)	0.043 (3)	0.0265 (18)	0.005 (2)	0.0019 (17)	−0.0077 (19)
C14	0.042 (2)	0.049 (3)	0.0241 (18)	0.006 (2)	−0.0105 (16)	−0.0080 (19)
C15	0.036 (2)	0.037 (3)	0.0248 (17)	0.0015 (18)	−0.0106 (15)	−0.0030 (18)
C16	0.039 (2)	0.035 (3)	0.033 (2)	−0.0027 (19)	−0.0084 (16)	−0.009 (2)
C17	0.032 (3)	0.053 (4)	0.024 (3)	0.000	−0.011 (2)	0.000
C18	0.045 (3)	0.049 (4)	0.018 (2)	0.000	0.002 (2)	0.000
C19	0.040 (2)	0.035 (3)	0.0304 (19)	−0.0059 (19)	0.0027 (16)	−0.0037 (19)
C20	0.049 (3)	0.086 (4)	0.035 (2)	−0.020 (3)	0.0105 (19)	−0.009 (3)
C21	0.037 (6)	0.100 (11)	0.024 (6)	−0.012 (7)	0.001 (4)	−0.003 (6)
C22	0.043 (5)	0.084 (9)	0.064 (6)	−0.014 (6)	0.024 (5)	−0.031 (6)
C23	0.046 (7)	0.084 (11)	0.093 (9)	0.009 (7)	0.008 (6)	0.014 (9)
C24	0.041 (6)	0.139 (15)	0.052 (7)	0.008 (8)	0.009 (5)	0.017 (9)
C25	0.070 (9)	0.33 (3)	0.039 (6)	0.079 (13)	−0.021 (6)	−0.056 (11)
C26	0.056 (6)	0.109 (10)	0.034 (5)	0.020 (6)	−0.020 (5)	−0.019 (6)
C21A	0.068 (8)	0.074 (9)	0.027 (6)	−0.007 (7)	0.006 (5)	−0.006 (6)
C22A	0.082 (8)	0.077 (9)	0.053 (6)	0.000 (7)	0.025 (5)	0.024 (6)
C23A	0.142 (15)	0.097 (13)	0.103 (11)	−0.053 (11)	0.025 (10)	0.034 (9)
C24A	0.069 (10)	0.18 (2)	0.097 (12)	−0.022 (12)	0.003 (9)	0.046 (15)
C25A	0.120 (17)	0.32 (4)	0.40 (5)	0.17 (2)	0.19 (3)	0.29 (4)
C26A	0.100 (12)	0.115 (15)	0.182 (19)	0.045 (11)	0.103 (13)	0.082 (14)
C27	0.037 (2)	0.039 (3)	0.044 (2)	−0.0059 (19)	−0.0069 (18)	−0.004 (2)
C28	0.047 (3)	0.045 (3)	0.099 (4)	−0.013 (2)	−0.001 (3)	−0.004 (3)
C29	0.049 (3)	0.032 (3)	0.086 (4)	−0.009 (2)	−0.005 (3)	0.007 (3)
C30	0.049 (3)	0.050 (3)	0.084 (4)	−0.012 (2)	−0.005 (3)	0.003 (3)
C31	0.043 (3)	0.064 (4)	0.113 (5)	−0.017 (3)	−0.007 (3)	0.008 (4)
C32	0.059 (4)	0.059 (4)	0.123 (6)	−0.016 (3)	0.016 (4)	0.022 (4)
C33	0.101 (5)	0.067 (5)	0.085 (4)	−0.014 (4)	0.013 (4)	0.022 (4)
C34	0.065 (3)	0.061 (4)	0.082 (4)	−0.008 (3)	−0.014 (3)	0.024 (3)
C35	0.046 (4)	0.039 (4)	0.079 (5)	0.000	0.013 (3)	0.000
C36	0.063 (5)	0.081 (7)	0.141 (9)	0.000	−0.022 (5)	0.000
C37	0.062 (5)	0.092 (7)	0.071 (5)	0.000	0.022 (4)	0.000
O1	0.0423 (15)	0.0252 (16)	0.0298 (13)	0.0013 (12)	−0.0057 (11)	0.0008 (12)
O2	0.0426 (15)	0.0283 (17)	0.0289 (12)	0.0066 (13)	−0.0055 (11)	0.0008 (12)
O3	0.0514 (16)	0.0368 (19)	0.0291 (13)	0.0033 (14)	0.0004 (12)	−0.0115 (13)
O4	0.0543 (17)	0.049 (2)	0.0240 (13)	0.0071 (15)	−0.0065 (12)	−0.0091 (14)
O5	0.052 (3)	0.067 (3)	0.073 (3)	0.000	0.002 (2)	0.000
Br1	0.0470 (3)	0.0497 (3)	0.0377 (2)	0.0141 (2)	0.00437 (17)	−0.0069 (2)
Br2	0.0349 (3)	0.0451 (4)	0.0703 (4)	0.000	−0.0152 (3)	0.000
Br3	0.0855 (5)	0.0737 (6)	0.0331 (3)	0.000	0.0234 (3)	0.000

### Geometric parameters (Å, °)

**Table:**

C1—C6	1.377 (5)	C20—H20C	0.9900
C1—C2	1.383 (5)	C20—H20D	0.9900
C1—Br1	1.897 (4)	C21—C22	1.3900
C2—O3	1.385 (4)	C21—C26	1.3900
C2—C3	1.391 (5)	C22—C23	1.3900
C3—C4	1.389 (5)	C22—H22	0.9500
C3—C16	1.540 (5)	C23—C24	1.3900
C4—C5	1.385 (5)	C23—H23	0.9500
C4—H4	0.9500	C24—C25	1.3900
C5—C6	1.381 (5)	C24—H24	0.9500
C5—C10	1.531 (5)	C25—C26	1.3900
C6—O1	1.397 (4)	C25—H25	0.9500
C7—O1	1.410 (5)	C26—H26	0.9500
C7—O2	1.422 (5)	C21A—C22A	1.3900
C7—H7A	0.9900	C21A—C26A	1.3900
C7—H7B	0.9900	C22A—C23A	1.3900
C8—C9	1.382 (5)	C22A—H22A	0.9500
C8—O2	1.385 (5)	C23A—C24A	1.3900
C8—C12	1.389 (5)	C23A—H23A	0.9500
C9—C11	1.394 (5)	C24A—C25A	1.3900
C9—C10	1.526 (5)	C24A—H24A	0.9500
C10—C19	1.535 (5)	C25A—C26A	1.3900
C10—H10	1.0000	C25A—H25A	0.9500
C11—C9^i^	1.394 (5)	C26A—H26A	0.9500
C11—H11	0.9500	C27—C28	1.494 (6)
C12—C8^i^	1.389 (5)	C27—H27A	0.9900
C12—Br2	1.882 (5)	C27—H27B	0.9900
C13—O4	1.395 (5)	C28—C29	1.520 (7)
C13—O3	1.411 (5)	C28—H28A	0.9900
C13—H13A	0.9900	C28—H28B	0.9900
C13—H13B	0.9900	C29—C34	1.357 (7)
C14—O4	1.388 (5)	C29—C30	1.413 (7)
C14—C15	1.393 (5)	C30—C31	1.368 (7)
C14—C18	1.394 (5)	C30—H30	0.9500
C15—C17	1.391 (5)	C31—C32	1.383 (9)
C15—C16	1.520 (6)	C31—H31	0.9500
C16—C27	1.529 (5)	C32—C33	1.375 (9)
C16—H16	1.0000	C32—H32	0.9500
C17—C15^i^	1.391 (5)	C33—C34	1.428 (8)
C17—H17	0.9500	C33—H33	0.9500
C18—C14^i^	1.394 (5)	C34—H34	0.9500
C18—Br3	1.880 (5)	C35—O5	1.204 (7)
C19—C20	1.526 (5)	C35—C37	1.470 (10)
C19—H19A	0.9900	C35—C36	1.544 (11)
C19—H19B	0.9900	C36—H36A	0.9800
C20—C21A	1.491 (7)	C36—H36B	0.9800
C20—C21	1.569 (7)	C37—H37A	0.9800
C20—H20A	0.9900	C37—H37B	0.9800
C20—H20B	0.9900		
			
C6—C1—C2	121.0 (3)	C19—C20—H20D	108.2
C6—C1—Br1	119.5 (3)	H20C—C20—H20D	107.4
C2—C1—Br1	119.5 (3)	C22—C21—C26	120.0
C1—C2—O3	119.7 (3)	C22—C21—C20	121.5 (5)
C1—C2—C3	119.7 (3)	C26—C21—C20	118.2 (5)
O3—C2—C3	120.6 (3)	C21—C22—C23	120.0
C4—C3—C2	118.1 (3)	C21—C22—H22	120.0
C4—C3—C16	122.1 (3)	C23—C22—H22	120.0
C2—C3—C16	119.7 (3)	C22—C23—C24	120.0
C5—C4—C3	122.5 (4)	C22—C23—H23	120.0
C5—C4—H4	118.7	C24—C23—H23	120.0
C3—C4—H4	118.7	C25—C24—C23	120.0
C6—C5—C4	118.1 (3)	C25—C24—H24	120.0
C6—C5—C10	119.0 (3)	C23—C24—H24	120.0
C4—C5—C10	122.8 (3)	C26—C25—C24	120.0
C1—C6—C5	120.5 (3)	C26—C25—H25	120.0
C1—C6—O1	118.6 (3)	C24—C25—H25	120.0
C5—C6—O1	120.8 (3)	C25—C26—C21	120.0
O1—C7—O2	112.8 (3)	C25—C26—H26	120.0
O1—C7—H7A	109.0	C21—C26—H26	120.0
O2—C7—H7A	109.0	C22A—C21A—C26A	120.0
O1—C7—H7B	109.0	C22A—C21A—C20	123.3 (7)
O2—C7—H7B	109.0	C26A—C21A—C20	116.4 (7)
H7A—C7—H7B	107.8	C21A—C22A—C23A	120.0
C9—C8—O2	121.3 (4)	C21A—C22A—H22A	120.0
C9—C8—C12	120.5 (4)	C23A—C22A—H22A	120.0
O2—C8—C12	118.1 (3)	C22A—C23A—C24A	120.0
C8—C9—C11	118.6 (4)	C22A—C23A—H23A	120.0
C8—C9—C10	118.4 (3)	C24A—C23A—H23A	120.0
C11—C9—C10	122.9 (3)	C23A—C24A—C25A	120.0
C9—C10—C5	106.6 (3)	C23A—C24A—H24A	120.0
C9—C10—C19	114.6 (3)	C25A—C24A—H24A	120.0
C5—C10—C19	114.1 (3)	C26A—C25A—C24A	120.0
C9—C10—H10	107.0	C26A—C25A—H25A	120.0
C5—C10—H10	107.0	C24A—C25A—H25A	120.0
C19—C10—H10	107.0	C25A—C26A—C21A	120.0
C9^i^—C11—C9	121.7 (5)	C25A—C26A—H26A	120.0
C9^i^—C11—H11	119.1	C21A—C26A—H26A	120.0
C9—C11—H11	119.1	C28—C27—C16	112.8 (4)
C8—C12—C8^i^	120.1 (5)	C28—C27—H27A	109.0
C8—C12—Br2	119.9 (2)	C16—C27—H27A	109.0
C8^i^—C12—Br2	119.9 (2)	C28—C27—H27B	109.0
O4—C13—O3	113.2 (3)	C16—C27—H27B	109.0
O4—C13—H13A	108.9	H27A—C27—H27B	107.8
O3—C13—H13A	108.9	C27—C28—C29	111.8 (4)
O4—C13—H13B	108.9	C27—C28—H28A	109.2
O3—C13—H13B	108.9	C29—C28—H28A	109.2
H13A—C13—H13B	107.8	C27—C28—H28B	109.2
O4—C14—C15	120.8 (4)	C29—C28—H28B	109.2
O4—C14—C18	119.5 (3)	H28A—C28—H28B	107.9
C15—C14—C18	119.6 (4)	C34—C29—C30	118.8 (5)
C17—C15—C14	118.2 (4)	C34—C29—C28	121.2 (5)
C17—C15—C16	121.7 (4)	C30—C29—C28	119.9 (5)
C14—C15—C16	120.1 (4)	C31—C30—C29	121.3 (5)
C15—C16—C27	112.9 (4)	C31—C30—H30	119.3
C15—C16—C3	106.4 (3)	C29—C30—H30	119.3
C27—C16—C3	113.8 (3)	C30—C31—C32	119.5 (5)
C15—C16—H16	107.8	C30—C31—H31	120.3
C27—C16—H16	107.8	C32—C31—H31	120.3
C3—C16—H16	107.8	C33—C32—C31	120.8 (6)
C15^i^—C17—C15	122.9 (5)	C33—C32—H32	119.6
C15^i^—C17—H17	118.5	C31—C32—H32	119.6
C15—C17—H17	118.5	C32—C33—C34	119.2 (6)
C14^i^—C18—C14	121.3 (5)	C32—C33—H33	120.4
C14^i^—C18—Br3	119.4 (2)	C34—C33—H33	120.4
C14—C18—Br3	119.4 (2)	C29—C34—C33	120.3 (5)
C20—C19—C10	111.3 (3)	C29—C34—H34	119.8
C20—C19—H19A	109.4	C33—C34—H34	119.8
C10—C19—H19A	109.4	O5—C35—C37	120.9 (7)
C20—C19—H19B	109.4	O5—C35—C36	118.9 (7)
C10—C19—H19B	109.4	C37—C35—C36	120.2 (7)
H19A—C19—H19B	108.0	C35—C36—H36A	108.7
C21A—C20—C19	116.3 (5)	C35—C36—H36B	109.9
C19—C20—C21	112.2 (4)	H36A—C36—H36B	109.5
C19—C20—H20A	109.2	C35—C37—H37A	110.0
C21—C20—H20A	109.2	C35—C37—H37B	109.2
C19—C20—H20B	109.2	H37A—C37—H37B	109.5
C21—C20—H20B	109.2	C6—O1—C7	117.8 (3)
H20A—C20—H20B	107.9	C8—O2—C7	117.4 (3)
C21A—C20—H20C	108.2	C2—O3—C13	116.7 (3)
C19—C20—H20C	108.2	C14—O4—C13	116.6 (3)
C21A—C20—H20D	108.2		
			
C6—C1—C2—O3	−175.0 (4)	C15—C14—C18—Br3	178.7 (3)
Br1—C1—C2—O3	3.5 (5)	C9—C10—C19—C20	−67.1 (5)
C6—C1—C2—C3	2.1 (6)	C5—C10—C19—C20	169.7 (4)
Br1—C1—C2—C3	−179.3 (3)	C10—C19—C20—C21A	−162.2 (6)
C1—C2—C3—C4	−0.8 (6)	C10—C19—C20—C21	177.0 (5)
O3—C2—C3—C4	176.3 (4)	C21A—C20—C21—C22	−62.9 (15)
C1—C2—C3—C16	176.4 (4)	C19—C20—C21—C22	43.4 (8)
O3—C2—C3—C16	−6.5 (6)	C21A—C20—C21—C26	110.6 (18)
C2—C3—C4—C5	−1.3 (6)	C19—C20—C21—C26	−143.1 (5)
C16—C3—C4—C5	−178.5 (4)	C26—C21—C22—C23	0.0
C3—C4—C5—C6	2.2 (6)	C20—C21—C22—C23	173.3 (8)
C3—C4—C5—C10	178.7 (4)	C21—C22—C23—C24	0.0
C2—C1—C6—C5	−1.3 (6)	C22—C23—C24—C25	0.0
Br1—C1—C6—C5	−179.8 (3)	C23—C24—C25—C26	0.0
C2—C1—C6—O1	174.4 (3)	C24—C25—C26—C21	0.0
Br1—C1—C6—O1	−4.1 (5)	C22—C21—C26—C25	0.0
C4—C5—C6—C1	−0.8 (6)	C20—C21—C26—C25	−173.6 (8)
C10—C5—C6—C1	−177.5 (4)	C19—C20—C21A—C22A	85.6 (8)
C4—C5—C6—O1	−176.4 (3)	C21—C20—C21A—C22A	167.9 (19)
C10—C5—C6—O1	6.9 (5)	C19—C20—C21A—C26A	−88.3 (8)
O2—C8—C9—C11	175.2 (3)	C21—C20—C21A—C26A	−6.0 (13)
C12—C8—C9—C11	−0.6 (6)	C26A—C21A—C22A—C23A	0.0
O2—C8—C9—C10	−7.2 (5)	C20—C21A—C22A—C23A	−173.7 (8)
C12—C8—C9—C10	176.9 (4)	C21A—C22A—C23A—C24A	0.0
C8—C9—C10—C5	−85.6 (4)	C22A—C23A—C24A—C25A	0.0
C11—C9—C10—C5	91.8 (4)	C23A—C24A—C25A—C26A	0.0
C8—C9—C10—C19	147.2 (3)	C24A—C25A—C26A—C21A	0.0
C11—C9—C10—C19	−35.4 (5)	C22A—C21A—C26A—C25A	0.0
C6—C5—C10—C9	85.6 (4)	C20—C21A—C26A—C25A	174.1 (8)
C4—C5—C10—C9	−90.9 (4)	C15—C16—C27—C28	173.1 (4)
C6—C5—C10—C19	−146.9 (4)	C3—C16—C27—C28	−65.4 (5)
C4—C5—C10—C19	36.6 (5)	C16—C27—C28—C29	166.2 (4)
C8—C9—C11—C9^i^	−1.0 (7)	C27—C28—C29—C34	−88.0 (6)
C10—C9—C11—C9^i^	−178.5 (3)	C27—C28—C29—C30	90.1 (6)
C9—C8—C12—C8^i^	2.3 (7)	C34—C29—C30—C31	2.1 (8)
O2—C8—C12—C8^i^	−173.7 (3)	C28—C29—C30—C31	−176.1 (5)
C9—C8—C12—Br2	179.8 (3)	C29—C30—C31—C32	−2.3 (9)
O2—C8—C12—Br2	3.9 (5)	C30—C31—C32—C33	1.3 (10)
O4—C14—C15—C17	−176.2 (3)	C31—C32—C33—C34	−0.1 (10)
C18—C14—C15—C17	0.6 (6)	C30—C29—C34—C33	−0.8 (8)
O4—C14—C15—C16	6.0 (5)	C28—C29—C34—C33	177.3 (5)
C18—C14—C15—C16	−177.2 (4)	C32—C33—C34—C29	−0.2 (9)
C17—C15—C16—C27	32.1 (5)	C1—C6—O1—C7	100.6 (4)
C14—C15—C16—C27	−150.1 (3)	C5—C6—O1—C7	−83.7 (4)
C17—C15—C16—C3	−93.5 (4)	O2—C7—O1—C6	88.9 (4)
C14—C15—C16—C3	84.3 (4)	C9—C8—O2—C7	84.2 (4)
C4—C3—C16—C15	93.1 (4)	C12—C8—O2—C7	−99.9 (4)
C2—C3—C16—C15	−84.0 (4)	O1—C7—O2—C8	−88.9 (4)
C4—C3—C16—C27	−31.9 (5)	C1—C2—O3—C13	−99.3 (4)
C2—C3—C16—C27	150.9 (4)	C3—C2—O3—C13	83.6 (5)
C14—C15—C17—C15^i^	1.3 (7)	O4—C13—O3—C2	−91.8 (4)
C16—C15—C17—C15^i^	179.1 (3)	C15—C14—O4—C13	−83.1 (4)
O4—C14—C18—C14^i^	174.3 (3)	C18—C14—O4—C13	100.1 (5)
C15—C14—C18—C14^i^	−2.6 (8)	O3—C13—O4—C14	91.1 (4)
O4—C14—C18—Br3	−4.4 (6)		

Symmetry codes: (i) *x*, −*y*+1/2, *z*.
